# Epicardial and Visceral Adipose Tissue and Global Longitudinal Strain: A Review of Cardiac Imaging Insights in Subclinical Myocardial Dysfunction

**DOI:** 10.3390/nu18061009

**Published:** 2026-03-23

**Authors:** Marco Vicardi, Afshin Farzaneh-Far, Cristiano Fava, Luca Dalle Carbonare, Simone Romano

**Affiliations:** 1 Unit of General Medicine C, Medicine Department, University of Verona, 37134 Verona, Italy; 2 Division of Cardiology, Department of Medicine, Duke University, Durham, NC 27708, USA

**Keywords:** epicardial adipose tissue, visceral adipose tissue, global longitudinal strain, speckle-tracking echocardiography, subclinical cardiovascular disease, mediterranean diet

## Abstract

**Background:** Visceral adipose tissue (VAT) and epicardial adipose tissue (EAT) are increasingly recognized as relevant contributors to cardiometabolic alterations and subclinical myocardial dysfunction, independently of overall obesity. Their pathogenic role extends beyond simple fat accumulation, encompassing inflammatory activation, lipotoxicity, and altered myocardial metabolism. **Objective:** This narrative review synthesizes current evidence on the relationships between VAT/EAT and myocardial strain parameters, with emphasis on subclinical cardiovascular risk detection and nutritional interventions. **Methods:** We conducted a comprehensive review of studies published between 2003–2025, focusing on imaging-based assessments of adipose tissue distribution and strain parameters using echocardiography, computed tomography, and cardiac magnetic resonance. **Results:** Increased EAT and, to a lesser extent, VAT showed significant associations with impaired global longitudinal strain (GLS) across imaging-based studies. In patients with type 2 diabetes, VAT mediated a substantial proportion of the association between insulin resistance and left ventricular dysfunction. Mediterranean diet adherence was associated with lower epicardial adipose tissue burden, while higher EAT was associated with persistent atrial fibrillation among patients with atrial fibrillation undergoing catheter ablation. Speckle-tracking echocardiography consistently showed superior prognostic value compared to ejection fraction for detecting subclinical dysfunction. **Conclusions:** VAT and EAT represent important mechanistic links between body composition and early myocardial dysfunction, identifiable through advanced strain imaging before clinical disease becomes apparent. These findings support the integration of multimodal cardiac imaging and nutritional interventions into cardiovascular prevention strategies, providing novel opportunities for early risk stratification and personalized treatment approaches.

## 1. Introduction

### 1.1. The Paradigm Shift in Cardiovascular Risk Assessment

Contemporary cardiovascular medicine is progressively moving beyond traditional risk factor-based approaches toward more refined phenotyping strategies that integrate imaging biomarkers and body composition analysis [1]. This evolution reflects growing recognition that standard risk calculators, while valuable, may incompletely reflect the complexity of individual cardiovascular risk profiles, particularly in metabolically heterogeneous populations.

The limitations of body mass index (BMI) as a cardiovascular risk predictor have become increasingly apparent, with substantial evidence demonstrating that adipose tissue distribution patterns provide superior prognostic information compared to total body weight measures [2]. This recognition has catalyzed intensive research into “ectopic fat” depots—adipose tissue accumulations in non-traditional locations that exert profound metabolic and cardiovascular effects through proximity-dependent and systemic mechanisms.

Visceral adipose tissue (VAT) and epicardial adipose tissue (EAT) have emerged as particularly relevant components of cardiovascular risk assessment and as potential targets of preventive strategies [1,3]. Unlike subcutaneous fat, these visceral depots exhibit heightened metabolic activity and inflammatory potential, directly influencing cardiovascular pathophysiology through paracrine, vasocrine, and systemic pathways [1]. The anatomical proximity of these fat depots to cardiac structures creates unique opportunities for both pathogenic interactions and therapeutic targeting.

### 1.2. Rationale for Focus on VAT and EAT

Visceral adipose tissue, defined as intra-abdominal fat surrounding internal organs, has been extensively studied for its role in metabolic syndrome and cardiovascular disease [4]. VAT demonstrates distinct characteristics compared to subcutaneous fat, including enhanced lipolytic activity, increased inflammatory cytokine production, and direct portal circulation connections that bypass first-pass hepatic metabolism. These properties position VAT as a critical mediator of insulin resistance, dyslipidemia, and systemic inflammation [4,5].

Epicardial adipose tissue represents an even more anatomically intimate cardiovascular risk factor [1,2]. Located between the myocardium and visceral pericardium, EAT shares microcirculation with the underlying myocardium and lacks fascial boundaries that would limit paracrine interactions [1]. This unique anatomical arrangement enables direct tissue-to-tissue communication through local release of adipokines, inflammatory mediators, and vasoactive substances [1].

The physiological functions of EAT under normal conditions include thermogenesis, mechanical cardiac protection, and metabolic buffering [1,6]. However, pathological EAT expansion and inflammatory activation transform this protective tissue into a source of cardiotoxic mediators that are associated with coronary atherosclerosis, myocardial fibrosis, and electrical remodeling [7,8].

### 1.3. Strain Imaging as Early Marker of Dysfunction

Speckle-tracking echocardiography (STE) has revolutionized the detection of subclinical cardiovascular dysfunction by enabling quantitative assessment of myocardial deformation parameters that precede changes in ejection fraction [9,10]. Global longitudinal strain (GLS) has emerged as the most robust and clinically validated strain parameter, consistently demonstrating superior sensitivity for detecting early systolic dysfunction and providing incremental prognostic value across diverse patient populations [9,10].

GLS has been consistently associated with prognostic outcomes across a wide spectrum of cardiovascular conditions and selected non-cardiovascular diseases, as demonstrated using multimodal cardiac imaging approaches, including echocardiography and cardiac magnetic resonance [11,12,13,14,15,16,17,18,19,20,21,22,23,24].

The technical advancement from tissue Doppler-based strain measurements to speckle-tracking methodologies has substantially improved reproducibility and clinical applicability [10,25]. Contemporary strain analysis can detect subtle alterations in myocardial mechanics that occur months to years before conventional echocardiographic abnormalities become apparent, creating unprecedented opportunities for early intervention and prevention [10].

Strain imaging applications extend beyond left ventricular assessment to include the evaluation of atrial mechanics [26]. Left atrial strain, in particular, has gained recognition as a sensitive marker of elevated filling pressures and predictor of atrial fibrillation development, with direct relevance to patients with increased visceral adiposity [26].

### 1.4. Review Objectives and Scope

This comprehensive review synthesizes current evidence linking VAT and EAT to myocardial strain abnormalities, with particular emphasis on subclinical cardiovascular risk detection and therapeutic implications. Our analysis encompasses carefully selected studies spanning foundational research, clinical investigations, and intervention trials published between 2003 and 2025.

The review addresses several critical knowledge gaps in the field: first, the comparative importance of VAT versus EAT in predicting strain abnormalities; second, the mechanistic pathways linking adipose tissue dysfunction to myocardial performance; third, the clinical utility of combined imaging approaches for risk stratification; and fourth, the evidence supporting nutritional and lifestyle interventions for modifying both adipose tissue characteristics and strain parameters.

**Figure 1:**
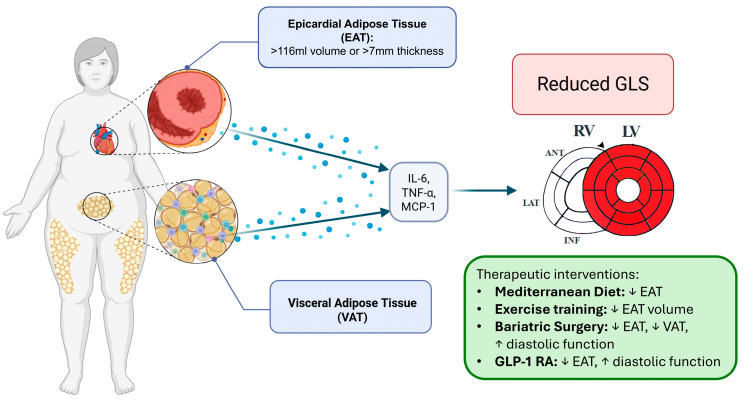
Conceptual overview of the association between epicardial and visceral adipose tissue and subclinical myocardial dysfunction. Increased epicardial adipose tissue (EAT) and visceral adipose tissue (VAT) are associated with a pro-inflammatory profile that correlates with impaired myocardial deformation, expressed as reduced left ventricular global longitudinal strain (GLS), highlighted in red in the figure. Lifestyle and weight-loss interventions are associated with reductions in EAT/VAT and parallel improvements in myocardial strain. Arrows (↑, ↓) indicate increase or decrease, respectively.

Special attention is devoted to nutritional interventions, reflecting growing interest in dietary pattern modifications as first-line therapeutics for cardiovascular prevention [23,24]. The Mediterranean diet, in particular, has demonstrated significant effects on both adipose tissue biology and cardiovascular outcomes, making it highly relevant for integrated prevention strategies that combine imaging biomarkers with lifestyle modifications [23,24] (Figure 1).

## 2. Adipose Tissue Biology and Cardiovascular Pathophysiology

### 2.1. Visceral Adipose Tissue: Beyond Energy Storage

Visceral adipose tissue represents far more than a passive energy storage depot, functioning as a highly active endocrine organ that profoundly influences cardiovascular health through multiple interconnected pathways [4,5,27]. The anatomical distribution of VAT encompasses intra-abdominal fat surrounding the liver, pancreas, and intestines, with direct venous drainage into the portal circulation, delivering adipose-derived mediators directly to the liver and influencing systemic metabolism through hepatic pathways [4,5].

At the cellular level, VAT adipocytes undergo progressive hypertrophy with lipid overload, transitioning toward a predominantly unilocular morphology with enlarged lipid droplets—a hallmark of dysfunctional white adipose tissue associated with mechanical stress, hypoxia, and impaired lipolytic regulation [28]. As adipocytes enlarge beyond a critical size, cellular stress and death increase, and recruited macrophages surround necrotic adipocytes forming characteristic crown-like structures (CLSs), a histological signature of inflamed visceral fat [29]. This macrophage infiltration is driven in part by adipocyte-derived MCP-1/CCL2, which recruits circulating monocytes that differentiate locally into pro-inflammatory M1-polarized macrophages—a phenotypic shift from the resident anti-inflammatory M2-like macrophages predominant in lean adipose tissue [30].

The inflammatory secretome of VAT includes a complex array of cytokines, chemokines, and adipokines that collectively promote cardiovascular dysfunction [27,31]. Key inflammatory mediators include tumor necrosis factor-alpha (TNF-α), interleukin-6 (IL-6), monocyte chemoattractant protein-1 (MCP-1), and C-reactive protein (CRP). In patients with type 2 diabetes, VAT demonstrates positive associations with insulin resistance and negative associations with left ventricular GLS (reflecting worse myocardial deformation) [5,27].

The mechanistic link between VAT-derived inflammation and impaired myocardial deformation is multifactorial. VAT contributes to insulin resistance through chronic secretion of pro-inflammatory cytokines and adipokines that impair insulin signaling pathways, combined with high lipolytic activity releasing free fatty acids (FFAs) and pro-inflammatory mediators into the systemic circulation [27]. Insulin resistance, in turn, exacerbates endothelial dysfunction, promotes atherogenesis, and leads to myocardial remodeling [27]. In cross-sectional evidence from patients with type 2 diabetes, VAT showed an independent positive association with LV-GLS (β = 0.482, 95% CI: 0.060–0.904, *p* = 0.039) after adjustment for multiple confounders; a causal mediation analysis revealed that VAT accounted for approximately 60.9% of the total effect of insulin resistance on LV-GLS [27]. This estimate derives from a single cross-sectional study using bioelectrical impedance for VAT quantification, with a wide confidence interval for the mediated proportion (95% CI: 15.82–171), reflecting the methodological uncertainty inherent to mediation analyses in limited samples [27]; confirmation in independent longitudinal cohorts is needed before generalization.

Enhanced FFA availability alters myocardial substrate utilization, shifting from glucose toward fatty acid oxidation and reducing cardiac efficiency [32,33]. Simultaneously, chronic inflammation promotes coronary endothelial dysfunction, microvascular rarefaction, and interstitial fibrosis that collectively impair myocardial mechanics [27,32,34].

### 2.2. Epicardial Adipose Tissue: The Heart’s Local Fat Depot

Epicardial adipose tissue possesses unique anatomical and functional characteristics that distinguish it from other visceral fat depots and create particularly intimate cardiovascular interactions [1,31]. The absence of fascial barriers between EAT and the underlying myocardium enables direct paracrine signaling, while the shared microcirculation facilitates bidirectional communication between adipocytes and cardiomyocytes, a feature that reflects the absence of a fascial barrier between EAT and the myocardium [1].

At the cellular and morphological level, EAT is a white adipose tissue with distinct brown fat-like and beige fat-like features that set it apart from both typical white adipose tissue and VAT [1,2]. In neonates, EAT is morphologically and functionally similar to brown adipose tissue, characterized by multilocular adipocytes and expression of uncoupling protein-1 (UCP-1). With ageing, the proportion of brown adipocytes progressively decreases in favour of larger unilocular white adipocytes, reflecting a functional transition from thermogenesis toward energy storage—a brown-to-beige transition that is a distinctive feature of EAT not shared by other visceral fat depots, which lack significant UCP-1 expression [1,35]. In pathological conditions such as coronary artery disease, this transition is further accelerated: the expression of genes encoding brown fat activation proteins is downregulated in EAT, with reciprocal increases in pro-inflammatory and pro-fibrotic mediators [1].

Under physiological conditions, EAT serves several protective functions including thermogenesis through brown adipose tissue-like activity, mechanical cushioning during cardiac contraction, and metabolic buffering that provides local energy substrates during periods of high cardiac demand [1,6,36]. The expression of uncoupling proteins and presence of multilocular adipocytes in healthy EAT support its thermogenic capacity and potential cardioprotective role [6,36].

The transition from protective to pathological EAT is triggered by a constellation of converging factors. Systemic obesity and metabolic syndrome drive EAT expansion beyond its buffering capacity, inducing adipocyte hypertrophy that promotes local hypoxia, a well-characterized stimulus for adipose tissue fibrosis and insulin resistance [1,4]. Local hypoxia activates hypoxia-inducible factor-1α (HIF-1α), which promotes adipose tissue fibrosis and insulin resistance through upregulation of collagen crosslinking enzymes such as lysyl oxidase, with an associated increase in local inflammation [37]. Macrophage infiltration into pathological EAT represents a critical step in phenotypic conversion: the shift from resident anti-inflammatory M2-like macrophages toward pro-inflammatory M1-polarized macrophages drives the local release of TNF-α, IL-6, and IL-1β, while simultaneously suppressing adiponectin secretion [1,7,31]. In patients with advanced coronary artery disease, dense macrophage infiltrates within EAT are a histological signature of this pathological conversion [1,31]. Furthermore, fibrosis and adipocyte apoptosis—common in end-stage cardiac disease—further alter the cellular composition of EAT and contribute to its dysfunction [1].

However, pathological EAT expansion transforms this beneficial tissue into a source of cardiotoxic mediators [7,31,36]. Inflammatory activation of EAT leads to increased production of pro-inflammatory cytokines including TNF-α, IL-6, and IL-1β, while simultaneously reducing protective adipokine secretion such as adiponectin [7,31]. This inflammatory shift creates a local environment that promotes coronary atherosclerosis, myocardial fibrosis, and electrical remodeling [7].

Regional heterogeneity within EAT adds additional complexity to its cardiovascular effects [1]. Pericoronary EAT demonstrates distinct pro-inflammatory characteristics with enhanced expression of inflammatory markers in regions adjacent to coronary atherosclerotic plaques [1,38]. Peri-atrial EAT is specifically implicated in atrial fibrillation pathogenesis through local fibrosis, adipocyte infiltration into atrial myocardium, and electrophysiological remodeling [39,40]. This spatial relationship suggests that EAT may both contribute to and respond to local coronary pathology, creating self-reinforcing cycles of inflammation and dysfunction [1,38].

### 2.3. Convergent Pathways to Myocardial Strain Impairment

The mechanistic pathways described separately for VAT and EAT converge on a final common effector: impaired myocardial mechanics detectable through strain parameters before changes in ejection fraction become apparent [32,34]. Alongside the inflammatory and insulin-resistance-mediated mechanisms outlined in the preceding sections, lipotoxicity represents an additional and direct pathway: excessive free fatty acid delivery from both VAT and EAT overwhelms myocardial oxidative capacity, leading to intramyocardial ceramide and diacylglycerol accumulation, mitochondrial uncoupling, and impaired excitation-contraction coupling that reduces contractile protein calcium sensitivity and promotes cardiomyocyte apoptosis [32,33,41,42]. These functional and ultrastructural changes contribute to reduced peak systolic strain values before any detectable alteration in left ventricular volumes or ejection fraction occurs [42,43].

Coronary microvascular dysfunction represents a further shared mechanism independent of epicardial coronary obstruction [8,44]. Inflammatory mediators derived from both VAT and EAT—including TNF-α, IL-6, and oxidized lipids—promote endothelial dysfunction, reduce nitric oxide bioavailability, and impair coronary flow reserve, generating heterogeneous regional myocardial perfusion that may be reflected in segmental strain abnormalities even in the absence of obstructive coronary artery disease [8,32,44]. This microvascular pathway may be particularly relevant in patients with metabolic syndrome and preserved ejection fraction, where regional strain heterogeneity often precedes global GLS impairment [44].

The temporal progression from metabolic stress to structural changes follows predictable patterns that can be monitored through serial strain assessments [43]. Early changes include reduced strain rate parameters reflecting impaired diastolic relaxation, followed by reductions in peak systolic strain values, and finally the development of regional strain heterogeneity that reflects evolving fibrosis patterns [43]. This predictable sequence creates opportunities for intervention through serial strain assessments before irreversible structural changes occur.

## 3. Imaging Modalities for Adipose Tissue Quantification and Technical Foundations of Speckle-Tracking Echocardiography

### 3.1. Imaging Modalities for Adipose Tissue Quantification

The accurate quantification of adipose tissue depots is a prerequisite for interpreting the clinical evidence linking VAT and EAT to myocardial strain. Each imaging modality offers distinct advantages and limitations, and the choice of technique has direct implications for the comparability of findings across studies.

Echocardiography remains the most widely used method for EAT assessment in clinical practice, owing to its low cost, wide availability, and absence of ionizing radiation. EAT thickness is measured as the echo-free space between the outer wall of the myocardium and the visceral layer of the pericardium, most reliably obtained perpendicularly on the right ventricular free wall at end-systole from the parasternal long-axis view, with the aortic annulus used as an anatomic landmark [2]. A threshold of ≥5 mm has been proposed as a marker of increased cardiometabolic risk [45]. However, echocardiographic EAT measurement provides a single linear dimension that may incompletely reflect total EAT volume and regional distribution; MDCT demonstrates superior sensitivity for measuring fat in the deeper atrioventricular and interventricular groove regions not accessible by transthoracic echocardiography [45].

Cardiac computed tomography (CT) enables volumetric quantification of EAT by delineating fat-containing voxels within defined attenuation thresholds—typically between −45 and −195 Hounsfield units—within the pericardial sac [1]. Both contrast-enhanced and non-contrast-enhanced cardiac-gated multidetector CT protocols are used; differences in CT attenuation values exist depending on the presence of iodinated contrast and on local inflammatory status, since EAT density is a recognized marker of adipose tissue inflammation [1]. CT additionally enables regional EAT characterization: pericoronary EAT can be interrogated through the fat attenuation index (FAI), a quantitative marker of perivascular adipose tissue inflammation that reflects transcriptomic, metabolic, and phenotypic changes in perivascular fat and is significantly higher around culprit coronary lesions compared with non-culprit lesions [46]. Peri-atrial EAT can be independently segmented and CT-derived posterior left atrial adiposity, including peri-atrial EAT thickness, is associated with atrial fibrillation burden independently of left atrial area and BMI [1,47]. For VAT, CT cross-sectional area at the L4–L5 vertebral level or volumetric abdominal CT represent the established reference standards [1].

Cardiac magnetic resonance (CMR) is considered the gold standard for volumetric EAT assessment, exploiting the high signal intensity of fat on T1-weighted sequences without ionizing radiation exposure [48]. CMR uniquely permits simultaneous assessment of myocardial tissue characterization—including extracellular volume fraction as a validated surrogate of interstitial fibrosis, and late gadolinium enhancement—enabling direct correlation of adipose tissue burden with structural myocardial changes within a single examination [4]. Feature-tracking strain analysis from CMR cine sequences further allows EAT volume and myocardial deformation parameters to be quantified in the same study, a methodological advantage of particular relevance for mechanistic research [4].

Cardiac magnetic resonance feature-tracking (CMR-FT) represents an increasingly used complementary approach for strain quantification, deriving deformation parameters directly from standard steady-state free precession cine sequences without requiring additional dedicated acquisitions [49]. However, while intra- and inter-observer reproducibility of cardiac magnetic resonance feature-tracking within individual software platforms is generally excellent, the principal unresolved standardization challenge for CMR-FT remains inter-vendor variability: the software vendor has been identified as a significant independent confounder of LV GLS values across studies, with pooled normal values differing systematically between CVI, Medis, and TomTec platforms, necessitating vendor-specific reference ranges and precluding direct cross-platform numerical comparisons [50].

A critical consideration when interpreting the clinical literature is that echocardiographic EAT thickness and CT/CMR-derived EAT volume are not interchangeable metrics [51]. Meta-analytic data demonstrate significant heterogeneity in EAT-outcome associations depending on the measurement method employed, with a 1 mm increase in echocardiographic EAT thickness representing a clinically very different change from a 1 mL increase in CT-derived EAT volume [51]. Volumetric assessments by CT and CMR generally provide stronger and more consistent associations with adverse cardiovascular outcomes, while echocardiographic thickness measurements, though more widely available, carry greater methodological variability [45,51]. Similarly, CT- and CMR-based adipose tissue quantification lacks a unified methodological standard: attenuation thresholds for EAT segmentation vary across studies (typically −195 to −45 HU for CT), volumetric measurements are not interchangeable with echocardiographic linear thickness, and no consensus protocol currently exists for peri-atrial or pericoronary fat segmentation [1,51]. 

These converging sources of methodological heterogeneity—across both strain imaging platforms and adipose tissue quantification techniques—must be considered when comparing results across the studies reviewed in the subsequent sections.

### 3.2. Physics and Technical Principles of Speckle-Tracking Echocardiography

Speckle-tracking echocardiography represents a significant advancement in cardiac imaging capability, enabling quantitative assessment of myocardial deformation [8,9,43]. The fundamental physics underlying STE involves analysis of frame-to-frame displacement of tissue speckles created by constructive and destructive interference patterns from ultrasound reflection at the tissue interface level [9,43,52].

Contemporary STE systems employ advanced pattern recognition algorithms that can track individual speckle patterns with high spatial resolution, depending on image quality and vendor-specific algorithms, enabling detection of minute tissue displacements that correspond to myocardial deformation [25,43,52]. The transition from one-dimensional tissue Doppler-based strain measurements to two-dimensional speckle tracking has dramatically improved accuracy by accounting for complex cardiac motion patterns including translation, rotation, and through-plane movement [43,52].

Strain parameters quantify myocardial deformation relative to the original tissue dimensions, typically expressed as percentage change from baseline length or area [10,25,52]. Longitudinal strain measures shortening along the long axis of the left ventricle, circumferential strain quantifies circumferential shortening around the ventricular circumference, and radial strain reflects thickening in the radial direction [52]. Each strain component provides unique insights into different aspects of myocardial function and pathology [52].

Advanced strain parameters including global longitudinal strain (GLS), strain rate, and myocardial work indices have demonstrated particular clinical utility for cardiovascular risk assessment [11,12,13,14,15,16,17,18,19,20,21,22,23,24,51]. GLS represents the average longitudinal deformation across all left ventricular segments and has emerged as the most robust and reproducible strain parameter for clinical use [52]. Strain rate parameters provide additional information about the velocity of myocardial deformation and have particular relevance for diastolic function assessment [52].

### 3.3. Standardization and Reproducibility of Speckle-Tracking Echocardiography

The development of consensus guidelines by the European Association of Cardiovascular Imaging (EACVI) and American Society of Echocardiography (ASE) has been crucial for improving the reliability and clinical applicability of STE when performed according to standardized protocols [9]. These guidelines provide detailed recommendations for image acquisition, analysis protocols, and quality control measures that ensure consistent results across different operators, laboratories, and vendor platforms [9].

Image acquisition standardization encompasses multiple technical considerations including frame rate optimization (50–80 fps for optimal tracking), depth and sector width adjustment to maximize spatial resolution, and gain settings that optimize speckle definition without introducing artifacts [9,10,53]. The guidelines emphasize the importance of obtaining high-quality images with clear endocardial definition, minimal artifacts, and complete myocardial visualization throughout the cardiac cycle [9,53].

Inter-vendor variability represents an ongoing challenge in STE implementation, with different manufacturers employing distinct tracking algorithms and reference standards that can produce systematic differences in strain measurements [25,54]. While absolute strain values may vary between vendors, the clinical utility of strain measurements for detecting abnormalities and tracking changes over time remains robust across platforms when appropriate vendor-specific reference ranges are applied [25,54].

Quality control measures include assessment of tracking quality indicators, manual correction of endocardial contours when necessary, and exclusion of segments with poor tracking quality from global strain calculations [9]. The guidelines recommend that global strain values should only be reported when adequate tracking quality is achieved in at least 12 of 16 left ventricular segments, ensuring that calculated values accurately represent true myocardial function [9].

### 3.4. Clinical Validation and Prognostic Value of Speckle-Tracking Echocardiography

Extensive clinical validation studies have consistently demonstrated the superior prognostic value of strain parameters compared to conventional echocardiographic measurements [11,12,13,14,15,16,17,18,19,20,21,22,23,24,51]. GLS has shown particular strength as a predictor of cardiovascular events, with meta-analyses demonstrating significant prognostic value across diverse patient populations including those with preserved ejection fraction, heart failure, and ischemic heart disease [11,12,13,14,15,16,17,18,19,20,21,22,23,24,51].

Comparison with other advanced imaging modalities including cardiac magnetic resonance (CMR) has demonstrated that while CMR remains the gold standard for myocardial tissue characterization, STE provides clinically comparable sensitivity for detecting regional dysfunction, with greater practicality for serial assessment in routine clinical settings [43].

The prognostic superiority of GLS over ejection fraction reflects its ability to detect subclinical dysfunction before chamber remodeling and obvious wall motion abnormalities develop [11,12,13,14,15,16,17,18,19,20,21,22,23,24,43]. This enhanced sensitivity is particularly relevant in patients with metabolic disorders and increased visceral adiposity, where early detection of myocardial dysfunction can guide preventive interventions before irreversible changes occur [24,43].

## 4. Clinical Evidence: VAT and EAT Relationships with Strain

### 4.1. EAT and Left Ventricular Strain Relationships

A growing body of evidence supports inverse associations between epicardial adipose tissue burden and left ventricular strain parameters across diverse patient populations, though the strength, independence, and prognostic implications of these associations vary substantially across studies depending on the population examined, the imaging modality used for EAT quantification, and the covariates included in multivariable models [3,7,8,55].

A systematic review and meta-analysis of 22 studies using volumetric EAT quantification demonstrated that EAT was significantly greater in patients with diastolic dysfunction compared with those with normal diastolic function (weighted mean difference 24.4 mL; 95% CI 18.5–30.4; *p* < 0.001), and was an independent predictor of impaired e′ and E/e′ after adjustment for age, sex, and measures of adiposity, whereas associations with LVEF were inconsistent and not independent of confounders [56].

Among the most methodologically rigorous investigations, Ng et al. examined 130 patients without obstructive coronary artery disease undergoing cardiac computed tomography and three-dimensional speckle-tracking echocardiography, demonstrating that EAT volume was the strongest independent predictor of impaired global longitudinal strain (standardized β = 0.512, *p* < 0.001), as well as circumferential, radial, and area strain components, even after adjustment for body mass index, waist to hip ratio, which was not independently associated with strain in the same model [3]. Importantly, this differential predictive performance of EAT over systemic obesity measures in a selected population without obstructive CAD or established cardiomyopathy supports the primacy of local paracrine mechanisms, though direct generalization to broader clinical populations requires caution.

The relationship between EAT and multidirectional strain components reveals differential effects on various aspects of myocardial mechanics [3,7]. While longitudinal strain shows the strongest and most reproducible associations with EAT burden, circumferential and radial strain correlations demonstrate greater variability across cohorts [3,57]. At the mechanistic level, Ng et al. demonstrated in a CMR-based study that increased EAT volume index was independently associated with both greater myocardial fat content and higher interstitial fibrosis burden quantified by extracellular volume fraction, suggesting that structural—not only inflammatory—pathways mediate GLS impairment [56,57].

Advanced strain parameters provide additional insights. In a CMR-based study of 225 metabolic syndrome patients, EAT was independently associated with impaired global longitudinal peak strain, and that reduced myocardial energetic efficiency index partially mediated approximately one quarter of this association, implicating metabolic uncoupling as an intermediate mechanism beyond inflammation alone [55]. In parallel, the relationship between EAT thickness and myocardial work indices has been demonstrated in metabolic syndrome cohorts using pressure-strain loop analysis, where thicker EAT was associated with higher global wasted work and lower global work efficiency, further supporting the concept of metabolic inefficiency as a pathway linking epicardial fat to subclinical dysfunction [7].

The independence of EAT-strain associations from conventional cardiovascular risk factors has been demonstrated in several studies, with EAT volume or thickness remaining significantly associated with GLS after adjustment for age, BMI, and metabolic covariates [3,57]. However, this should not be interpreted as evidence that EAT acts in isolation: the available studies vary substantially in their covariate adjustment sets and populations, and the mechanistic primacy of anatomical proximity over systemic metabolic effects remains unresolved [55,58].

Strain parameters demonstrate superior sensitivity to subclinical myocardial dysfunction compared to conventional indices such as ejection fraction, detecting impairment before overt functional deterioration becomes apparent [58]. The prognostic implications of the EAT–strain relationship extend beyond cross-sectional associations: in the Framingham Heart Study cohort (n = 1554), increased EAT thickness was independently associated with incident heart failure, and mediation analysis demonstrated that impaired GLS partially mediated this effect on HF risk [59]. Consistent with this, in a prospective multicenter study of patients with HFmrEF and HFpEF, CMR-derived EAT volume was independently associated with the composite of all-cause mortality and HF hospitalization (HR 1.76 per SD, 95% CI 1.24–2.50, *p* = 0.001), with patients with high EAT volume exhibiting lower GLS [60]. Cardiovascular outcomes in these populations are nonetheless shaped by multiple interacting clinical and biological determinants, and the available evidence does not support framing EAT-strain parameters as having established superiority over conventional clinical risk models in unselected populations.

Taken together, the available evidence supports a biologically plausible and clinically detectable association between EAT burden and LV GLS impairment, mediated through local inflammatory, structural, metabolic, and neurohumoral mechanisms. Significant heterogeneity in study populations, imaging methodologies, and adjustment strategies currently limits the ability to define universal quantitative thresholds or establish definitive prognostic hierarchies, representing a key knowledge gap for future prospective, methodologically harmonized studies.

### 4.2. VAT and Myocardial Function Relationships

Visceral adipose tissue exerts its effects on myocardial mechanics primarily through systemic, rather than local paracrine, mechanisms—a fundamental distinction from EAT that has direct implications for understanding population-level associations and for identifying therapeutic targets. VAT is a metabolically active depot that promotes chronic low-grade inflammation through excess secretion of pro-inflammatory adipokines, drives insulin resistance, and activates neurohormonal pathways including the renin–angiotensin–aldosterone system, each of which independently affects myocardial energetics and deformation [27].

The most direct evidence of a quantitative contribution of VAT to myocardial strain impairment comes from Martinez-Dominguez et al., who conducted a sophisticated mediation analysis in 195 patients with type 2 diabetes and demonstrated that VAT mediates approximately 60.9% of the relationship between insulin resistance and left ventricular global longitudinal strain [27]. This mediation effect positions VAT as a key mechanistic intermediate linking metabolic dysfunction to subclinical cardiac dysfunction, rather than a mere contributor. It also distinguishes the VAT–strain relationship from the more direct paracrine pathway operative for EAT. Importantly, when EAT and VAT are examined in the same multivariable model, their relative contributions to GLS impairment are partially dissociated: whereas EAT volume independently predicts GLS even after adjustment for BMI and waist-to-hip ratio [3], the VAT–GLS relationship is substantially attenuated when systemic insulin resistance is included as a covariate, consistent with its role as a mediator rather than an independent anatomical risk factor [27].

Myocardial mechano-energetic efficiency (MEE)—estimated as the stroke volume-to-heart rate ratio indexed to LV mass, a non-invasive surrogate of the balance between myocardial work and energy consumption—provides a mechanistically informative intermediate between VAT burden and strain impairment. In 480 subjects with increased BMI without known cardiovascular disease enrolled in the FATCOR Study, reduced MEE index was independently associated with lower LV systolic function in both the longitudinal (GLS) and circumferential direction, after adjustment for LV mass, age, sex, hypertension, and triglyceride levels [61]. This relationship is amplified in the context of metabolic syndrome and insulin resistance: MEE declines progressively with increasing degrees of insulin resistance, and low MEE is a strong independent predictor of adverse cardiovascular outcomes in hypertensive patients with LV hypertrophy [62]. Taken together, these findings suggest that VAT-mediated insulin resistance impairs myocardial energetic efficiency, which in turn translates into measurable strain deterioration—a pathway that is mechanistically distinct from, though potentially additive to, the direct paracrine inflammatory cascade attributable to EAT.

Population-based evidence allows for better contextualization of the relationship between ectopic fat depots and LV remodeling. In 997 Framingham Heart Study participants undergoing CT and CMR, pericardial fat, intrathoracic fat, and VAT were all correlated with LV mass and left atrial dimension, although these associations were not independent of other adiposity measures, with the exception of pericardial fat and left atrial dimension in men—findings that underscore the difficulty of disentangling individual fat depot contributions in the setting of correlated systemic adiposity [63]. The cross-sectional design of this analysis limits causal inference, and the absence of strain measurements in that cohort precludes direct comparison with studies employing GLS as the primary outcome measure.

The mechanistic and clinical differentiation between VAT and EAT contributions to myocardial strain impairment has meaningful implications for risk stratification and therapeutic strategy. EAT acts predominantly through direct paracrine delivery of pro-inflammatory mediators to the adjacent myocardium, with its anatomical proximity conferring a degree of independence from systemic adiposity markers [3,57]; VAT, by contrast, exerts its influence through systemic metabolic pathways—principally insulin resistance, dyslipidaemia, and neurohormonal activation—that are modifiable through both lifestyle interventions and pharmacological therapies [27]. This distinction suggests that risk stratification strategies relying exclusively on global adiposity indices such as BMI may underestimate the cardiometabolic risk attributable to VAT in metabolically dysregulated individuals with preserved or near-normal body weight.

The degree to which VAT reduction translates into measurable improvement in myocardial strain parameters represents an important open question with direct clinical relevance. The cross-sectional design of the available studies precludes definitive conclusions regarding the efficacy of VAT-targeted interventions on myocardial strain recovery [27,63]. Nevertheless, the finding that VAT mediates approximately 60.9% of the insulin resistance–GLS relationship in type 2 diabetes [27] provides a pathophysiological rationale for prioritizing strategies that address visceral adiposity and insulin resistance—rather than body weight alone—in the cardiovascular risk management of metabolically dysregulated patients. Prospective interventional studies are needed to establish whether VAT reduction translates into measurable GLS improvement.

### 4.3. Atrial Strain and Adipose Tissue Relationships

Left atrial strain parameters demonstrate particularly strong associations with both VAT and EAT, reflecting the intimate anatomical relationships between atrial tissue and surrounding adipose depots [26,64].

The pathophysiological basis for atrial strain-adipose tissue relationships involves several interconnected mechanisms including direct inflammatory effects on atrial myocardium, promotion of atrial fibrosis through pro-fibrotic mediator release, and alteration of atrial electrophysiological properties [34,39]. EAT surrounding the left atrium demonstrates particularly intense inflammatory activity in patients with atrial fibrillation, creating local environments that promote arrhythmia initiation and perpetuation [34,39].

Atrial strain parameters serve as sensitive markers of atrial myopathy that precede the development of clinically apparent atrial fibrillation [26,39,64]. Studies utilizing comprehensive atrial strain analysis demonstrate that patients with increased EAT exhibit reduced atrial reservoir function, impaired conduit function, and enhanced booster pump function as compensatory responses [26,39,64].

The relationship between visceral adiposity and atrial strain extends beyond structural changes to include metabolic and autonomic effects [34,65]. Increased VAT is associated with enhanced sympathetic activity, altered atrial electrophysiological properties, and increased susceptibility to triggered activity, all of which contribute to atrial strain abnormalities and arrhythmia risk [34,65].

Therapeutic implications of atrial strain-adipose tissue relationships are particularly relevant for atrial fibrillation prevention and management [23]. The main clinical studies investigating the relationship between epicardial or visceral adipose tissue and myocardial strain are summarized in Table 1.

## 5. Nutritional and Therapeutic Interventions and Strain Outcomes

### 5.1. Mediterranean Diet Evidence

The Mediterranean diet represents the most extensively studied dietary pattern for cardiovascular prevention in the context of adipose tissue biology [53,54]. Evidence for a direct effect on epicardial adiposity comes from the PREDIMAR substudy, in which 199 patients with atrial fibrillation undergoing catheter ablation were enrolled: higher Mediterranean diet adherence (MEDAS score ≥ 7) was independently associated with lower odds of elevated EAT (≥135 g) on CT (multivariable OR 0.45; 95% CI 0.22–0.91; *p* = 0.025), and EAT ≥ 135 g was in turn significantly associated with persistent atrial fibrillation at baseline (multivariable OR 2.22; 95% CI 1.03–4.79; *p* = 0.042) [23]. The mechanistic basis for these observations includes the anti-inflammatory properties of polyphenols and monounsaturated fatty acids present in extra-virgin olive oil and nuts [68,69], and the broader cardiovascular benefit of the Mediterranean dietary pattern was established by the PREDIMED trial, in which supplementation with extra-virgin olive oil or nuts significantly reduced incident cardiovascular events [69].

### 5.2. Weight Loss Interventions, Bariatric Surgery, and Emerging Therapeutic Approaches

A systematic review and meta-analysis by Launbo et al. confirmed that exercise training, dietary intervention, bariatric surgery, and pharmacological therapies each produce significant reductions in EAT volume, with the largest effect sizes observed for bariatric surgery [70]. Consistent with this, a meta-analysis by Pereira et al. encompassing 35 studies demonstrated that EAT decreased significantly in 21 of 22 studies following bariatric surgery, with a large pooled effect size (ES = −0.89; 95% CI −1.23 to −0.55; *p* < 0.001) [67]. The functional cardiac correlates of EAT reduction have been documented prospectively: a study evaluating weight loss through caloric restriction showed that reductions in epicardial adipose tissue were accompanied by improvements in cardiac structure and function at follow-up [71]. With respect to differential procedural effects, a nationwide Swiss cohort study of 39,067 patients followed for up to 11 years demonstrated that Roux-en-Y gastric bypass was associated with a lower risk of MACE compared to sleeve gastrectomy (HR 0.75; 95% CI 0.64–0.88), driven primarily by a reduction in acute myocardial infarction [72].

Beyond surgical approaches, a randomized CMR study demonstrated that structured weight management in overweight patients with atrial fibrillation produced a significant reduction in pericardial fat volume and left atrial dimensions compared with controls at 12 months [66].

### 5.3. Pharmacological Approaches and Mechanistic Basis of Cardioprotection

**Figure 2:**
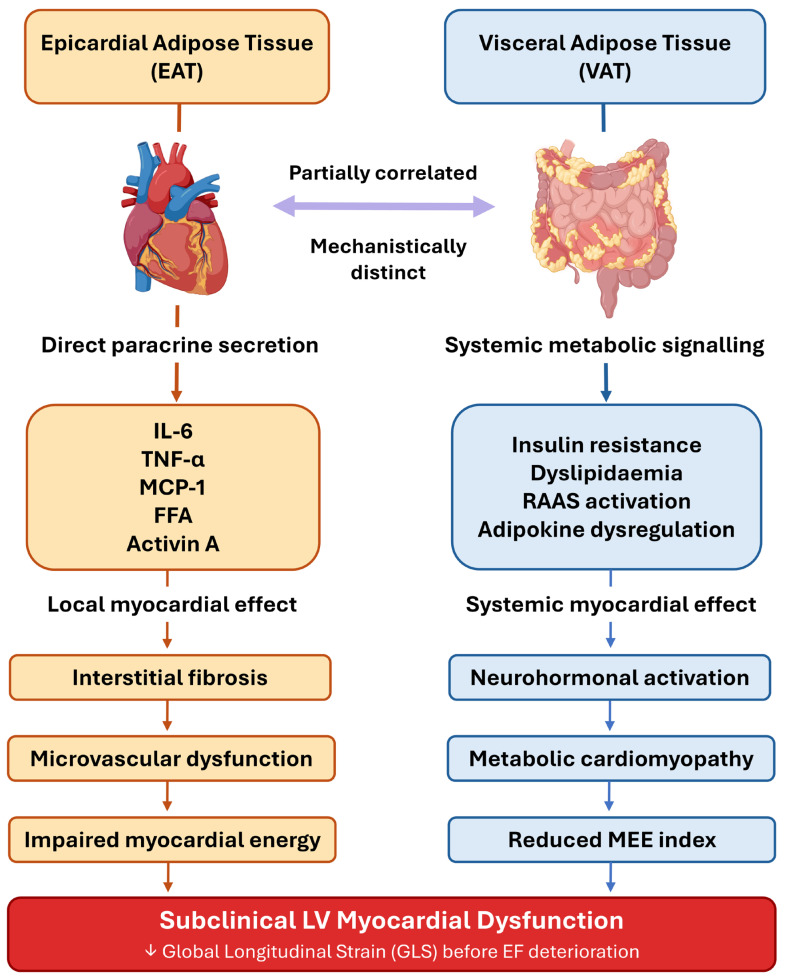
Mechanistic pathways linking epicardial adipose tissue (EAT) and visceral adipose tissue (VAT) to impaired left ventricular global longitudinal strain (GLS). Both pathways converge on subclinical myocardial dysfunction detectable before ejection fraction (EF) deterioration. FFA, free fatty acids; IL-6, interleukin-6; MCP-1, monocyte chemoattractant protein-1; MEE, myocardial energy efficiency; RAAS, renin–angiotensin–aldosterone system; TNF-α, tumour necrosis factor-alpha. The downward arrow (↓) indicates a reduction in GLS.

The cardioprotective effects of nutritional and pharmacological interventions in the context of excess adipose tissue operate through partially overlapping mechanistic axes. Reductions in EAT volume attenuate the paracrine inflammatory burden on the adjacent myocardium—mediated principally by TNF-α, IL-6, and MCP-1—while reductions in VAT mass reduce systemic low-grade inflammation, improve insulin sensitivity, and mitigate the chronic metabolic milieu driving myocardial lipotoxicity and interstitial fibrosis [1,36] (Figure 2). Bariatric surgery produces the most substantial and rapid reductions in both EAT and VAT, accompanied by significant improvements in CRP, PAI-1, and adiponectin, consistent with a broad metabolic reset of adipose tissue biology [38]. GLP-1 receptor agonists exert a partly direct effect on EAT through the preferential expression of GLP-1R in epicardial—but not subcutaneous—adipose tissue; receptor activation reduces local adipogenesis, improves free fatty acid oxidation, induces adipocyte browning, and modulates the renin–angiotensin–aldosterone system [1].

Among pharmacological approaches, GLP-1 receptor agonists have been the most extensively studied. A meta-analysis of 15 randomized trials (n = 898) found that GLP-1 receptor agonists significantly improved diastolic function as measured by the E/e’ ratio, reduced left ventricular mass and left atrial volume, and lowered NT-proBNP concentrations, without a significant effect on LVEF or GLS [73]. Preliminary mechanistic data from a small crossover study in eight adults with type 2 diabetes using combined GLP-1/glucagon receptor agonism demonstrated increased myocardial glucose uptake and a significant improvement in the left ventricular global peak diastolic circumferential strain rate, although GLS did not change significantly [74].

These findings are mechanistically coherent with the known vulnerability of subendocardial longitudinal fibres—the substrate of GLS—to ischaemia, inflammation, and interstitial fibrosis [75]. The haemodynamic effects of GLP-1 receptor agonists primarily relieve the conditions impairing ventricular relaxation and filling, explaining the preferential improvement in diastolic parameters. Recovery of longitudinal systolic strain likely requires more profound structural remodelling—including regression of interstitial fibrosis and restoration of subendocardial energetic integrity—processes that unfold over longer time horizons than those captured in existing trials, which have been predominantly short in duration and underpowered for strain endpoints.

## 6. Clinical Applications and Future Directions

### 6.1. Risk Stratification Strategies

Evidence from prospective and population-based studies supports the prognostic relevance of integrating adipose tissue imaging with myocardial strain assessment, potentially improving cardiovascular risk stratification beyond traditional risk factors. In the Framingham Heart Study cohort, increased EAT thickness was independently associated with incident heart failure, with impaired GLS identified as a partial mediator of this relationship [59]; in patients with HFmrEF and HFpEF, CMR-derived EAT volume independently predicted the composite of all-cause mortality and HF hospitalization, with higher EAT burden associated with lower GLS [60]. These findings directly translate the mechanistic pathways described before—local EAT-driven paracrine inflammation, structural fibrosis, and metabolic uncoupling—into prognostically meaningful endpoints.

Target populations for comprehensive imaging-based risk assessment include patients with metabolic syndrome, pre-diabetes, and obesity, particularly those with intermediate risk profiles where traditional algorithms provide limited guidance [27,76]. In these populations, GLS impairment may be detectable before ejection fraction deterioration [58], and VAT burden mediates a substantial proportion of the insulin resistance-GLS relationship [27], supporting early imaging-guided identification of individuals at elevated cardiac risk. The practical feasibility of this approach is enhanced by the availability of validated clinical surrogates for VAT—such as the Metabolic Score for Visceral Fat, a composite index incorporating the METS-IR insulin resistance score, waist-to-height ratio, age and sex, validated against DXA, MRI and bioimpedance—that do not require advanced body composition imaging [27,77].

### 6.2. Emerging Technologies and Research Directions

Artificial intelligence applications represent a promising avenue for advancing adipose tissue and strain assessment, particularly for automated analysis.

Three-dimensional and four-dimensional strain imaging technologies provide increasingly sophisticated assessments of myocardial mechanics that may reveal subtle abnormalities not detected by conventional two-dimensional approaches, as well as the regional EAT distribution across the atrioventricular and interventricular grooves [43]. However, robustness and inter-vendor comparability of 3D strain measurements remain suboptimal compared with two-dimensional GLS, and correct interpretation requires careful attention to loading conditions, ventricular geometry, and image quality [43]. Dedicated studies examining 3D strain specifically in the context of EAT and VAT burden are currently lacking.

Deep learning approaches for automated EAT segmentation from CT represent a clinically relevant advance, directly addressing the operator-dependent variability in manual EAT quantification discussed in Section 3. A deep learning network trained and validated on 3720 CCTA scans demonstrated excellent agreement with expert segmentation (CCC 0.970) and robust performance in technically challenging anatomies [78]; a separate multicenter algorithm reduced quantification time from approximately 15 min to under 2 s while maintaining expert-level accuracy across multiple scanner platforms [79]. Integration of such automated adipose tissue quantification with standardized strain analysis pipelines would enable the large-scale, reproducible datasets needed to establish population-level EAT–strain reference ranges and validate the prognostic thresholds suggested.

### 6.3. Implementation Science and Clinical Integration

The evidence reviewed in this manuscript supports a tiered approach to implementation. In secondary and tertiary care settings—where patients with metabolic syndrome, type 2 diabetes, or HFpEF/HFmrEF are routinely evaluated—the addition of EAT thickness measurement to standard echocardiographic examination represents a low-cost, zero-radiation increment to existing protocols, given that EAT can be measured from the parasternal long-axis view already acquired during routine study [2]. GLS assessment is increasingly available on contemporary echocardiographic platforms and is recommended by major imaging societies for subclinical dysfunction surveillance in high-risk metabolic populations [58]. The combined EAT–GLS protocol is therefore feasible within the framework of a standard echocardiographic examination without requiring dedicated additional imaging sessions.

In primary care and community settings, where CT or CMR are not routinely available, clinically validated VAT surrogates such as METS-VF—which requires only anthropometric measures and fasting metabolic indices—can identify patients with excess visceral adiposity and elevated insulin resistance who warrant referral for more detailed cardiac imaging [27,77]. This is particularly relevant in populations with intermediate cardiovascular risk where BMI alone provides insufficient discriminatory information [76].

The principal barriers to broader implementation remain the methodological heterogeneity in EAT quantification and strain measurement across vendors documented in Section 3, which currently prevents direct cross-institutional numerical comparisons and limits the transferability of risk thresholds across platforms [49,50]. Harmonization of acquisition and post-processing protocols, facilitated by the automated tools described above, is a prerequisite for translating the mechanistic and epidemiological insights reviewed here into standardized, guideline-endorsed clinical algorithms.

## 7. Conclusions

This narrative review examines the relationships between visceral and epicardial adipose tissue burden and impaired myocardial strain parameters, with a particular focus on left ventricular global longitudinal strain as a marker of subclinical cardiac dysfunction. The available evidence, while conceptually coherent, is derived predominantly from cross-sectional observational studies with considerable variability in effect sizes, adipose tissue quantification methods, and strain measurement platforms across cohorts. Methodological heterogeneity currently precludes direct numerical comparisons across studies and the establishment of universally applicable risk thresholds.

The mechanistic pathways linking adipose tissue dysfunction to myocardial strain impairment—including paracrine inflammatory signalling from EAT, VAT-mediated systemic insulin resistance, and downstream effects on myocardial energetics and interstitial fibrosis—represent biologically plausible hypotheses supported by convergent experimental and clinical data, but should not yet be considered fully established causal links.

The clinical translation of these findings is constrained by several important limitations: the absence of standardized protocols for adipose tissue quantification and strain assessment across imaging vendors; the lack of prospective outcome-driven trials with GLS as a pre-specified endpoint; and the absence of cost-effectiveness analyses for integrated EAT–strain screening strategies. Until such evidence is available, the relationships described here should be interpreted as hypothesis-generating rather than as the basis for definitive guideline recommendations.

From a clinical perspective, ventricular GLS impairment in the context of elevated EAT or VAT may represent an early signal of cardiomyopathic remodelling amenable to metabolic intervention, whereas left atrial strain abnormalities in this setting are more likely to reflect an atrial myopathy substrate with distinct implications for atrial fibrillation risk—a distinction with direct relevance to patient selection and therapeutic targeting. Integrating adipose tissue imaging with strain assessment holds clinical promise, particularly in metabolically dysregulated populations with intermediate cardiovascular risk, but requires extensive further research before broad clinical adoption can be justified.

## Figures and Tables

**Table 1:** Summary of Key Studies on Adipose Tissue and Myocardial Strain.

Study (Year)	Population	Imaging Modality	Key Findings
Ng et al. (2016) [3]	130 patients without obstructive CAD	CT + 3D STE	EAT volume strongest predictor of impaired 3D GLS (β = 0.512, *p* < 0.001), circumferential, radial, and area strain; independent of BMI and waist/hip ratio
Martinez-Dominguez et al. (2025) [27]	195 T2D patients	Echo + BIA	VAT mediates 60.9% of IR-GLS relationship; positive VAT-GLS association (β = 0.482, *p* = 0.039)
Choy et al. (2023) [59]	1554 participants, Framingham Heart Study	CMR	EAT thickness associated with incident HF (HR 1.43, 95% CI 1.18–1.73); NT-proBNP and GLS mediate the EAT-HF relationship
Barrio-Lopez et al. (2024) [23]	199 AF patients (PREDIMAR)	CT	Higher MedDiet adherence associated with lower EAT (mOR = 0.45, 95% CI 0.22–0.91); EAT ≥ 135 g associated with persistent AF
Chong et al. (2023) [51]	19,709 patients (meta-analysis)	CT + Echo	EAT associated with cardiac death (OR 2.53), MI (OR 2.63), AF (OR 4.04)
Sun et al. (2023) [7]	194 patients with suspected MetS	Echo	Increased EAT thickness was associated with alterations in myocardial work indices, including reduced global work efficiency, and with worse LV mechanical performance compared with subjects with thinner EAT
Nerlekar et al. (2018) [56]	22 studies (meta-analysis)	Various	EAT associated with diastolic dysfunction (WMD 24.43 mL); inverse correlation with LVEF inconsistent
Abed et al. (2015) [66]	87 AF patients	CMR	Weight management reduced pericardial fat (140.9 to 118.8 cm^3^) and LA volumes
Pereira et al. (2023) [67]	35 studies (meta-analysis)	Various	Bariatric surgery: significant EAT reduction; ES = −0.89, 95% CI: −1.23 to −0.55
Lobeek et al. (2025) [26]	82 HFmrEF/HFpEF patients	CMR	EAT independently associated with LA mechanical dysfunction (OR 2.85, 95% CI 1.04–7.79); 41% had LA dysfunction
Wang et al. (2025) [64]	113 HFpEF + 48 controls	Echo	EAT thickness greater in HFpEF (8.0 ± 1.0 vs. 5.0 ± 0.7 mm); inverse correlation with LA strain parameters (LASr, LAScd, LASct)

Abbreviations: AF, atrial fibrillation; BIA, bioelectrical impedance analysis; CMR, cardiac magnetic resonance; CT, computed tomography; EAT, epicardial adipose tissue; Echo, echocardiography; GLS, global longitudinal strain; HF, heart failure; HFpEF, heart failure with preserved ejection fraction; HFmrEF, heart failure with mildly reduced ejection fraction; HR, hazard ratio; IR, insulin resistance; LA, left atrium; LASr, left atrial strain reservoir; LAScd, left atrial strain conduit; LASct, left atrial strain contraction; MedDiet, Mediterranean diet; MetS, metabolic syndrome; MI, myocardial infarction; mOR, multivariable odds ratio; OR, odds ratio; STE, speckle-tracking echocardiography; WMD, weighted mean difference.

## Data Availability

No new data were created or analyzed in this study. Data sharing is not applicable to this article.
